# Genetic disease risks of under-represented founder populations in New York City

**DOI:** 10.1371/journal.pgen.1011755

**Published:** 2025-06-24

**Authors:** Mariko Isshiki, Anthony J. Griffen, Paul Meissner, Paulette Spencer, Michael D. Cabana, Susan D. Klugman, Mirtha Colón, Zoya Maksumova, Shakira Suglia, Carmen R. Isasi, John M. Greally, Srilakshmi M. Raj

**Affiliations:** 1 Departments of Genetics, Albert Einstein College of Medicine, Bronx, New York, United States of America; 2 Department of Biological Sciences, Graduate School of Science, The University of Tokyo, Tokyo, Japan; 3 Cell Biology, Albert Einstein College of Medicine, Bronx, New York, United States of America; 4 Family and Social Medicine, Albert Einstein College of Medicine, Bronx, New York, United States of America; 5 Obstetrics & Gynecology and Women’s Health, Albert Einstein College of Medicine, Bronx, New York, United States of America; 6 Bronx Community Health Network, Bronx, New York, United States of America; 7 Department of Pediatrics, Albert Einstein College of Medicine, Bronx, New York, United States of America; 8 Hondurans Against AIDS/Casa Yurumein, Bronx, New York, United States of America; 9 La Mesa, California, United States of America; 10 Department of Epidemiology, Rollins School of Public Health, Emory University, Atlanta, eorgia, United States of America; 11 Department of Epidemiology and Population Health, Albert Einstein College of Medicine, Bronx, New York, United States of America; Universitat Leipzig, GERMANY

## Abstract

The detection of founder pathogenic variants, those observed in high frequency only in a group of individuals with increased inter-relatedness, can help improve delivery of health care for that community. We identified 16 groups with shared ancestry, based on genomic segments that are shared through identity by descent (IBD), in New York City using the genomic data of 25,366 residents from the All Of Us Research Program and the Mount Sinai Bio*Me* biobank. From these groups we defined 7 as founder populations, mostly communities currently under-represented in medical genomics research, such as Puerto Rican and Garifuna. The enrichment analysis of ClinVar pathogenic or likely pathogenic (P/LP) variants in each group identified 201 of these damaging variants across the seven founder populations. We confirmed disease-causing variants previously reported to occur at increased frequencies in Ashkenazi Jewish and Puerto Rican genetic ancestry groups, but most of the damaging variants identified have not been previously associated with any such founder populations, and most of these founder populations have not been described to have increased prevalence of the associated rare disease. Twenty-two of 47 variants meeting Tier 2 prenatal screening criteria (1/100 carrier frequency within these founder groups) have never previously been reported. We show how population structure studies can provide insights into rare diseases disproportionately affecting under-represented founder populations, delivering a health care benefit but also a potential source of stigmatization of these communities, who should be part of the decision-making about implementation into health care delivery.

## Author summary

It is well recognized that genomic studies have been biased towards individuals of European ancestry, and that obtaining medical insights for populations under-represented in medical genomics is crucial to achieve health equity. Here, we use genomic information to identify networks of individuals in New York City who are distinctively related to each other, allowing us to define populations with common genetic ancestry based on genetic similarities rather than by self-reported race or ethnicity. In our study of >25,000 New Yorkers, we identified seven highly-interrelated founder populations, with 201 likely disease-causing variants with increased frequencies in specific founder populations. Many of these population-specific variants are new discoveries, despite their high frequency in founder populations. Studying recent genetic ancestry can help reveal population-specific disease insights that can help with early diagnosis, carrier screening, and opportunities for targeted therapies that all help to reduce health disparities in genomic medicine.

## Introduction

Rare diseases collectively occur in 3.5-5.9% of the population [1]. They involve significant morbidity and mortality, risk to family members and socio-economic consequences, and thus have the characteristics typical of a public health priority. Responding to this public health issue by studying rare diseases on a population scale is challenging because of the difficulty identifying individuals and families with uncommon conditions that are often refractory to diagnosis. An eventual solution will involve widespread application of sequencing of patients’ entire genomes in health care with sensitive and high-confidence prediction of damaging DNA sequence variants, but this remains a remote goal at present. In the interim, a typical approach in clinical practice is to use a person’s ‘genetic ancestry group’ [2] to highlight the rare diseases that are more common in that community and could be affecting the patient presenting for care. Populations that experienced small population size in the past tend to have enrichment for otherwise rare genetic conditions due to a ‘founder effect’, as exemplified in Ashkenazi Jewish individuals for their well-characterized set of genetic conditions [3,4]. By identifying other populations with founder effects, the genetic conditions more likely to occur in individuals from those communities can also be defined, and clinicians who serve these communities can be prepared to look out for these conditions. Extending the insights into rare disease risks for genetic ancestry groups other than White Europeans has been limited by the failure to include non-European populations in genomics research [5,6]. This bias magnifies health disparities and impedes effective delivery of medical care to marginalized groups and underserved populations. Recognizing this neglect, the All of Us (AoU) Research Program in the United States has been designed to represent the country’s diversity [7,8]. In this study, we focused on the genomes of individuals in New York City (NYC), representing a diverse and admixed urban population studied extensively through AoU as well as the separate Bio*Me* biobank [9,10]. We show how population genetics approaches using these data resources are able to reveal previously undiscovered rare disease susceptibilities in diverse genetic ancestry groups, particularly those with a founder effect. We were able to define groups with increased ‘genetic similarity’ [2] and characterize population structure by identifying segments of DNA that are shared among individuals due to inheritance from a common ancestor, also called identity-by-descent (IBD). This has previously been performed successfully in cohorts of different genetic ancestries [10–14] with implications for understanding population-specific disease risk [9,10,13]. Here we explicitly test the association between population structure and disease risk by focusing on population-specific enrichment of variants curated as disease-causing in the ClinVar database [15]. The results show how health systems and providers can benefit from recognizing rare diseases in the populations they serve, including the potential benefits of early detection of rare diseases as well as prenatal carrier screening in these communities, and the targeted use of specific therapies.

## Results

### The population structure of NYC participants of the all of us research program

**Fig 1:**
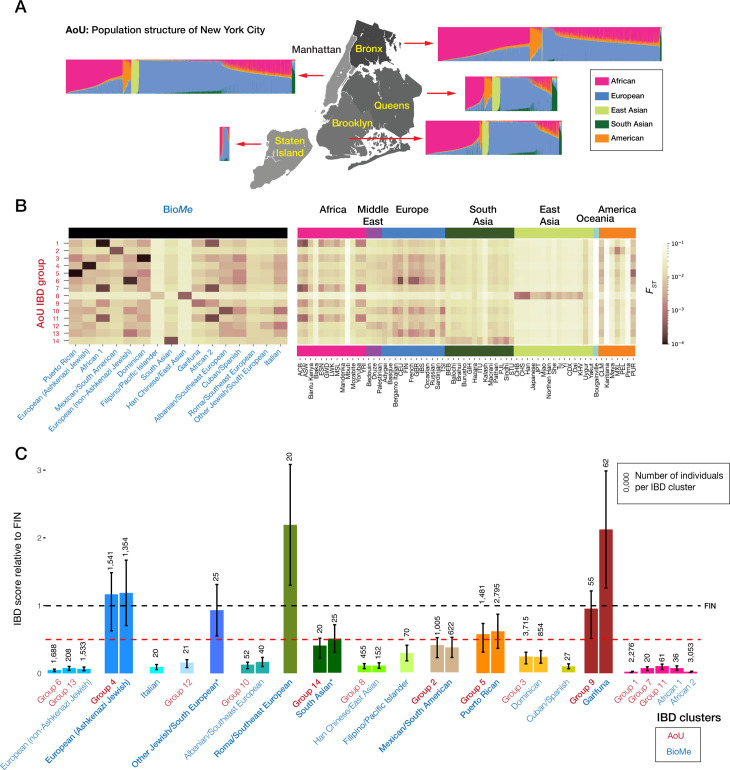
Population membership and geospatial distribution of All of Us IBD groups. (A) The geospatial distribution of all AoU individuals across NYC boroughs. This figure was generated in R using a shapefile obtained from: https://geodacenter.github.io/data-and-lab/nyc/; (B) Pairwise *F*_ST_ comparisons between AoU IBD groups, Bio*Me* IBD groups and global reference populations; (C) IBD scores for AoU and BioMe IBD groups relative to Finns in 1KGP (FIN), with sample sizes above each bar. IBD groups with *F*_ST_ < 0.001 are indicated by the same color, and founder population labels are in bold. The red dotted line indicates the IBD score for FIN and black dotted line indicates a relative IBD score of 0.5, which is the cut-off value we used to define a founder population (**Fig 2**); Only IBD groups with 20 and more individuals are shown to protect participants’ privacy based on the AoU Data and Statistics Dissemination Policy. An asterisk next to labels represents populations with inadequate reference information for annotation.

We studied the genetic diversity of NYC residents using genetic data from 13,817 participants of the AoU Research Program. This dataset excludes ‘related’ individuals, those who are second cousins or closer. We found that the AoU cohort in NYC showed different ancestry compositions across the five boroughs including evidence of substantial admixture in NYC participants (**Figs 1A and** S1), and that the proportions of self-reported race/ethnicity information for each borough are comparable to those from census data (S2A Fig) [16]. Of the boroughs, Manhattan and the Bronx are over-represented (S2B Fig).

We constructed a network based on IBD sharing to capture fine-scale recent population structure in the AoU NYC participants. After filtering edges to only reflect recent shared ancestry and exclude close familial ties, 98.6% of the cohort was included in the network. Among these individuals, we identified 14 IBD groups with a minimum of 20 individuals each (S1B Fig), representing 91% of the AoU NYC cohort. To allow comparison of our AoU results with results using the independent NYC Bio*Me* biobank, we repeated the IBD analysis similarly on Bio*Me.* The network included 95.6% of the Bio*Me* cohort. We identified 16 groups with ≥20 individuals representing 92.5% of the Bio*Me* cohort (**Fig 1B**), consistent with their published results [9,10]. We found that AoU and Bio*Me* have several similar populations with *F*_*ST*_ < 0.001 between them, even after removing related individuals across both datasets, reflecting their shared NYC recruitment area (**Fig 1B**).

### Detection of founder populations in NYC

**Fig 2:**
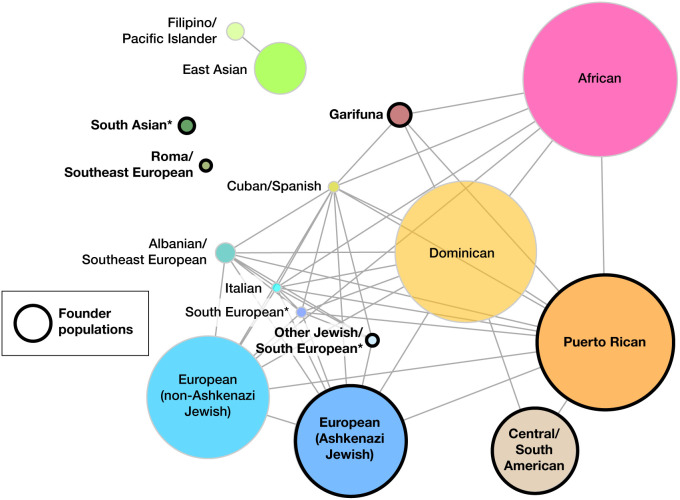
IBD groups identified in NYC from the combined AoU and Bio*Me* datasets. A network depiction of the IBD groups based on *F*_ST_ between IBD groups. Edges were weighted by negative logarithm of *F*_ST_. Only edges representing *F*_ST_ < 0.01 are shown, with founder populations circled in black. Circle sizes reflect the number of individuals in each IBD group. An asterisk next to labels represent populations with inadequate reference information for annotation.

**Fig 3:**
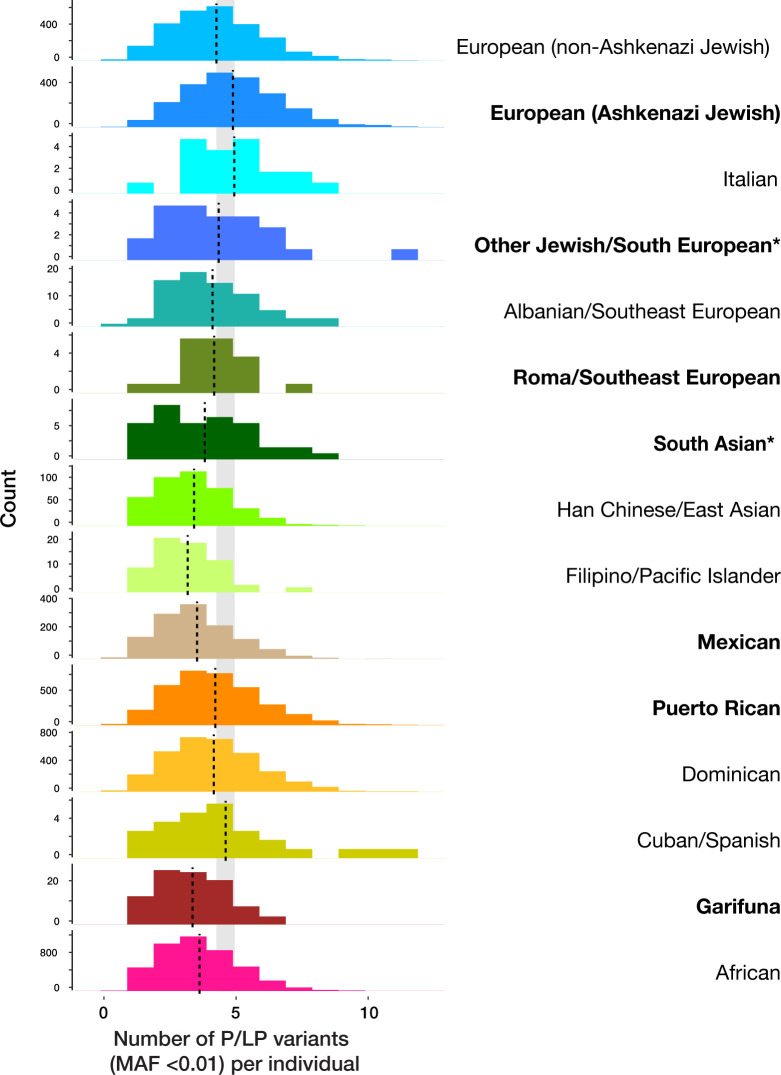
Number of P/LP variants per individual for each NYC IBD group identified from the AoU and Bio*Me* datasets. Each histogram shows the number of P/LP variants with minor allele frequency < 0.01 in AoU and Bio*Me* per individual for 15 IBD groups. The vertical dashed line indicates the mean value in each group. The ‘South European’ IBD group only included WGS data for fewer than 20 individuals and was removed due to the All of Us Data and Statistics Dissemination Policy. The shaded gray rectangle represents the range of mean values for the uppermost three European groups, highlighting the lower number of ClinVar variants annotated in those of Asian, American and African ancestries. Asterisk next to labels represent populations with inadequate reference information for annotation.

Of the IBD groups, there were five from AoU and seven from Bio*Me* with IBD scores >0.5, defining them as founder populations (**Fig 1C**). Of the seven founder groups from BioMe, founder effects in Ashkenazi Jewish, Puerto Rican and Garifuna were reported previously [9,10]. Since our AoU dataset and Bio*Me* are both NYC cohorts with shared genetic features (**Figs 1B**, **1C and** S3), we combined the IBD groups from AoU and Bio*Me* with pairwise Hudson’s *F*_*ST*_ values < 0.001, resulting in 16 IBD groups which we annotated based on inferred ancestry (**Fig 2**). Seven groups were identified as founder populations (IBD score >0.5). The populations were named based on self-defined ancestry as provided by the reference datasets, when available (**Figs 1B**, **1C**, **2 and 3 and** S1 Table). Genetic ancestry also acts on a continuum [17], therefore some IBD groups appeared to be more discrete (*e.g.,* Garifuna, Puerto Rican), whereas others include individuals across a broader geographic range (*e.g.,* Mexican/South American, Han Chinese/East Asian). In situations where groups could not be confidently labeled, the closest associated population group was used as a placeholder label until more genomic reference datasets become available. These populations are labeled with an asterisk (**Figs 1B**, **1C**, **2 and 3**).

### Identification of pathogenic founder variants

We then studied the genomes of the members of the founder populations to identify pathogenic variants characterizing each group. We used the ClinVar resource [15] as a curated source of disease-causing variants, while recognizing that the bias of ClinVar towards documentation of variants in individuals of European ancestry [18]. We focused on recurrent, rare, disease-causing variants, given our focus on founder effects. By requiring the pathogenic/likely pathogenic (P/LP) variant to occur in at least 2 individuals not from the same family, we identified 3,616 P/LP recurrent variants in NYC individuals. Consistent with the known ClinVar bias [18], European ancestry IBD groups showed more pathogenic variants than other IBD groups, especially when compared with individuals with African ancestry (P-value for Wilcoxon rank sum test between non-Ashkenazi Jewish European and African groups < 2.2 x 10^-16^) (**Fig 3**). This result suggests that the numbers of P/LP variants in non-European groups are underestimated, and that more disease-causing variants remain to be discovered in these understudied groups.

We detected 670 unique P/LP variants significantly enriched across the 7 founder populations (Fisher’s Exact *p *< 0.05) (S2 Table) with 201 of these variants passing Bonferroni correction. Of the 670 variants, 475 variants have two or more ClinVar gold stars, meaning variants are from practice guidelines, reviewed by expert panel, or from multiple submitters with evidence and no classification conflicts. Those variants with no ClinVar gold stars should in general be interpreted with caution, such as the *KRT18* variant not previously described to be common in Ashkenazi Jewish individuals. The *CD55* variant associated with protein-losing enteropathy [19] and shown in cell studies to cause loss of CD55 on the cell surface [20] also lacks a star rating, illustrating how the absence of this rating should not be used to exclude variants as disease-causing. This *CD55* variant also does not pass Bonferroni correction, nor does the known founder effect variant in *HBB* in the South Asian group [21,22], prompting us to include variants that do not pass multiple testing correction in **Table 1** as candidate founder disease-causing variants in the IBD groups. We identified 47 variants from this broader list that have minor allele frequencies of > 0.005 in one or more IBD groups, the Tier 2 threshold for inclusion into prenatal carrier screening panels [23]. Of these, 22 are new, previously unrecognized founder effect variants (**Table 1).**

**Table 1:** ClinVar P/LP variants with allele frequencies exceeding 1/200 within seven founder populations in NYC.

Gene	ClinVar accession	HGVS description	ClinVar star rating	Disease	Allele frequency (Bold: significant following Bonferroni correction)	Published founder variant (PMID)
**European (Ashkenazi Jewish)**						
*F11*	VCV000011892	NM_000128.4(F11):c.901T > C (p.Phe301Leu)	2	Hereditary factor XI deficiency	**0.0248**	2813350
*GJB2*	VCV000017010	NM_004004.6(GJB2):c.167del (p.Leu56fs)	3	Autosomal recessive nonsyndromic hearing loss	**0.0157**	9819448
*ELP1*	VCV000006085	NM_003640.5(ELP1):c.2204 + 6T > C	2	Familial dysautonomia	**0.0154**	12116234
*HEXA*	VCV000003889	NM_000520.6(HEXA):c.1274_1277dup (p.Tyr427fs)	2	Tay-Sachs disease	**0.0142**	2848800
*F11*	VCV000011891	NM_000128.4(F11):c.403G > T (p.Glu135Ter)	2	Hereditary factor XI deficiency	**0.0140**	2813350
*KRT18*	VCV000014585	NM_000224.3(KRT18):c.383A > T (p.His128Leu)	none	Cirrhosis	**0.0098**	Not described
*CFTR*	VCV000007129	NM_000492.4(CFTR):c.3846G > A (p.Trp1282Ter)	4	Cystic fibrosis	**0.0094**	1384328
*MPL*	VCV000135563	NM_005373.3(MPL):c.79 + 2T > A	2	Congenital amegakaryocytic thrombocytopenia	**0.0067**	21489838
*DDX11*	VCV000252749	NM_030653.4(DDX11):c.1763-1G > C	2	Warsaw breakage syndrome	**0.0063**	31287223
*ASPA*	VCV000002605	NM_000049.4(ASPA):c.854A > C (p.Glu285Ala)	2	Canavan disease	**0.0058**	8252036
*SLC3A1*	VCV000336195	NM_000341.4(SLC3A1):c.808C > T (p.Arg270Ter)	2	Cystinuria	**0.0054**	7539209
*FKTN*	VCV000003203	NM_001079802.2(FKTN):c.1167dup (p.Phe390fs)	2	Walker-Warburg congenital muscular dystrophy	**0.0054**	19266496
*CLRN1*	VCV000004395	NM_174878.3(CLRN1):c.144T > G (p.Asn48Lys)	2	Usher syndrome type 3A	**0.0052**	12145752
*PAH*	VCV000102706	NM_000277.3(PAH):c.506G > A (p.Arg169His)	3	Phenylketonuria	**0.0050**	29144512
*DLD*	VCV000011966	NM_000108.5(DLD):c.685G > T (p.Gly229Cys)	2	Pyruvate dehydrogenase E3 deficiency	**0.0050**	14765544
*FANCC*	VCV000012045	NM_000136.3(FANCC):c.456 + 4A > T	2	Fanconi anemia complementation group C	**0.0050**	8348157
Other Jewish/South European*						
*FMO3*	VCV000985096	NM_001002294.3(FMO3):c.1499G > A (p.Arg500Gln)	1	Trimethylaminuria	**0.0600**	Not described
*CD55*	VCV000431759	NM_000574.5(CD55):c.596C > T (p.Ser199Leu)	none	Complement hyperactivation, angiopathic thrombosis, and protein-losing enteropathy	0.0400	35314883
*KLKB1*	VCV000012033	NM_000892.5(KLKB1):c.337C > T (p.Arg113Ter)	none	Prekallikrein_deficiency	0.0400	Not described
**Puerto Rican**						
*HPS1*	VCV000005277	NM_000195.5(HPS1):c.1472_1487dup (p.His497fs)	2	Hermansky-Pudlak syndrome 1	**0.0097**	8896559
*RSPH4A*	VCV000088863	NM_001010892.3(RSPH4A):c.921 + 3_921 + 6del	2	Primary ciliary dyskinesia	**0.0096**	23798057
*COL27A1*	VCV000143245	NM_032888.4(COL27A1):c.2089G > C (p.Gly697Arg)	2	Steel syndrome	**0.0091**	24986830
*TBCK*	VCV000225235	NM_001163435.3(TBCK):c.376C > T (p.Arg126Ter)	2	Infantile hypotonia, infantile with psychomotor retardation and characteristic facies 3	**0.0089**	29283439
*ABCB4*	VCV000802326	NM_000443.4(ABCB4):c.2784-12T > C	1	Progressive familial intrahepatic cholestasis type 3	**0.0080**	34678161
*ERCC6L2*	VCV000421974	NM_020207.7(ERCC6L2):c.19C > T (p.Gln7Ter)	2	Bone marrow failure syndrome 2	**0.0076**	Not described
*MYO15A*	VCV000164548	NM_016239.4(MYO15A):c.7226del (p.Pro2409fs)	2	Deafness, autosomal recessive 3	**0.0063**	Not described
*LTBP2*	VCV001515466	NM_000428.3(LTBP2):c.2908 + 1G > A	1	Microspherophakia and/or megalocornea, with ectopia lentis and with or without secondary glaucoma	**0.0059**	Not described
*ECE1*	VCV000009133	NM_001397.3(ECE1):c.2260C > T (p.Arg754Cys)	none	Hirschsprung disease, cardiac defects, and autonomic dysfunction	**0.0056**	Not described
*MRPS34*	VCV000438633	NM_023936.2(MRPS34):c.322-10G > A	2	Leigh Syndrome	**0.0054**	28777931
*GDAP1*	VCV000004202	NM_018972.4(GDAP1):c.692C > T (p.Pro231Leu)	2	Charcot-Marie-Tooth disease	**0.0053**	34057104
*SGCG*	VCV000002009	NM_000231.3(SGCG):c.787G > A (p.Glu263Lys)	2	Limb-girdle muscular dystrophy type 2C	**0.0051**	16832103
**Roma/Southeast European**						
*TSEN54*	VCV000620188	NM_207346.3(TSEN54):c.1039A > T (p.Lys347Ter)	2	Pontocerebellar hypoplasia	**0.0526**	Not described
South Asian*						
*PAH*	VCV000092741	NM_000277.3(PAH):c.355C > T (p.Pro119Ser)	3	Phenylketonuria	**0.0385**	Not described
*ABCC2*	VCV000426249	NM_000392.5(ABCC2):c.3337del (p.Val1114fs)	2	Dubin-Johnson syndrome	0.0256	Not described
*HBB*	VCV000015437	NM_000518.5(HBB):c.92 + 1G > T	2	Beta thalassemia	0.0256	2064964
*CEP152*	VCV000158223	NM_001194998.2(CEP152):c.1155del (p.Thr386fs)	2	Primary microcephaly 9	0.0256	Not described
*TGFBI*	VCV001175370	NM_000358.3(TGFBI):c.1406G > A (p.Arg469His)	none	Granular corneal dystrophy	0.0256	Not described
*FANCE*	VCV001696377	NM_021922.3(FANCE):c.2_7del (p.Met1_Ala2del)	1	Fanconi anemia	0.0256	Not described
**Garifuna**						
*MYBPC3*	VCV000164113	NM_000256.3(MYBPC3):c.1484G > A (p.Arg495Gln)	2	Hypertrophic cardiomyopathy	**0.0245**	Not described
*DUOX2*	VCV000004065	NM_001363711.2(DUOX2):c.1126C > T (p.Arg376Trp)	2	Congenital hypothyroidism	**0.0196**	Not described
*CNGB1*	VCV001031963	NM_001297.5(CNGB1):c.1217G > A (p.Trp406Ter)	1	Retinitis pigmentosa	**0.0147**	Not described
*COL18A1*	VCV001484134	NM_001379500.1(COL18A1):c.2214 + 1G > A	1	Glaucoma, primary closed-angle; Knobloch syndrome	**0.0147**	Not described
*GBE1*	VCV000478912	NM_000158.4(GBE1):c.993-1G > T	2	Glycogen storage disease, type IV; Polyglucosan body disease, adult form	**0.0147**	Not described
*BBS12*	VCV000550386	NM_152618.3(BBS12):c.1151del (p.Ser384fs)	2	Bardet-Biedl syndrome 12	**0.0147**	Not described
*GALC*	VCV000429982	NM_000153.4(GALC):c.379C > T (p.Arg127Ter)	2	Krabbe disease	0.0098	Not described
*COL7A1*	VCV001454264	NM_000094.4(COL7A1):c.7244dup (p.Met2415fs)	2	Dystrophic epidermolysis bullosa	0.0098	Not described
*EOGT*	VCV000523593	NM_001278689.2(EOGT):c.78_81del (p.His27fs)	2	Adams-Oliver syndrome 4	0.0098	Not described

The table shows the P/LP variants that occur within each population at a frequency of ≥ 1/200 alleles (~1 in 100 individuals at a diploid locus), the threshold for inclusion in prenatal carrier screening [23]. Variants with allele frequencies in bold passed Bonferroni correction. Of the 47 variants, 25 have been published as founder variants, especially in the European (Ashkenazi Jewish) and Puerto Rican populations. The other 22 are new, previously unrecognized founder effect variants. Abbreviations: PMID, PubMed reference number. An asterisk next to labels represent populations with inadequate reference information for annotation. The AoU Resource Access Board reviewed this work and granted an exception to their Data and Statistics Dissemination policy to report frequencies representing <20 AoU participants.

### Ancestry analysis for shared founder variants in individuals of Caribbean ancestry

**Fig 4:**
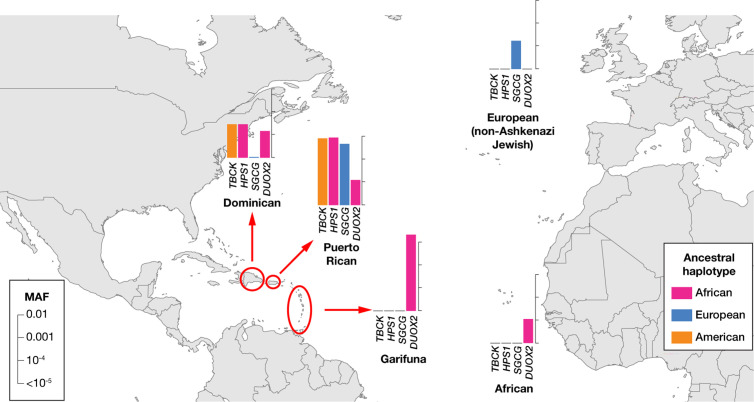
Shared founder variant frequencies in Caribbean IBD groups. Four examples of founder variants in Caribbean IBD groups are illustrated with comparisons of frequencies in other IBD groups within the NYC cohort, which includes AoU and Bio*Me*. Color indicates ancestral haplotypes inferred through local ancestry analysis. This map was generated using R libraries rworldmap and maps.

We detected 12 and 9 founder P/LP variants for Puerto Rican and Garifuna IBD groups, including variants that did not pass Bonferroni correction, respectively (**Table 1**). Of these 21 variants, 15 were also detected at lower frequencies in other IBD groups (S3 Table). To test whether this was due to shared ancestry, we inferred local ancestry (the origin of the DNA containing each P/LP variant) in the Caribbean individuals to identify the ancestral population in which each P/LP variant arose originally. We found the majority of the founder variants in Puerto Ricans to be located on haplotypes of European ancestry, with the remaining founder variants located on African and Indigenous American haplotypes (S3 Table). Of these, the origin of the *COL27A1* Steel syndrome variant on chromosome 9 has also previously been characterized as Native American ancestry [24]. We illustrate the sharing of founder effect variants across Caribbean groups and with European and African groups in **Fig 4**. Local ancestry analysis reveals an African origin of the *HPS1* and *DUOX2* pathogenic variants, an Indigenous American origin of the *TBCK* variant and a European origin of the *SGCG* pathogenic variants. All of these variants were not observed to be exclusive to one IBD group but occur across multiple Caribbean groups, reflecting the complex ancestries of these populations and the weakness of demographic categories such as race and ethnic origin as the sole predictors of genetic disease risks. The *MYBPC3* variant that occurs frequently in the Garifuna is on an African haplotype, but was also found in a European haplotype in an individual unassigned to any IBD group, which warrants further investigation into the ancestral origin of this variant in non-Garifuna. Local ancestry analysis can be used to reveal the evolutionary history of founder effect variants. The presence of a founder effect variant does not by itself indicate that a person is part of a known founder population.

## Discussion

In this study, we showed that several founder populations exist in the megacity of New York, and that the individuals from these genetic ancestry groups have distinctive increased risks of rare genetic diseases. The AoU Research Program [25] has deliberately included communities with genetic ancestries other than those who solely represent the non-Hispanic White demographic. We used genetic similarity as defined by the IBD sharing network and focused on DNA sequence variants with strong prior evidence for causing genetic diseases, to rediscover many known rare disease-causing variants common in the better-studied Ashkenazi Jewish and Puerto Rican communities, while revealing new founder variants in these and other founder populations in NYC. This recognition of variants enriched in founder populations is for clinically evaluating a patient’s rare disease diagnosis, as well as including these variants and conditions in prenatal carrier screening. In the Ashkenazi Jewish community, genetic testing panels for prenatal use is expanding to include presymptomatic testing for conditions affecting the parents [26]. The American College of Medical Genetics has recommended that “carrier screening paradigms should be ethnic and population neutral and more inclusive of diverse populations to promote equity and inclusion” [27]. This study demonstrates how current carrier screening panels can be expanded to serve a broader set of under-represented communities, using the example of the diverse population of NYC.

We used IBD sharing network analysis to identify groups of individuals who share genetic similarities in an unbiased manner [10–14]. However, this method of identifying groups is not without limitations. Varying the length thresholds of IBD segments, using different community detection algorithms, or combining or annotating IBD groups based on different *F*_ST_ thresholds will all change the resolution of population structure that is captured. The assignment of individuals to a group within the IBD sharing network is also highly dependent on who is included in the network. There may be individuals who lie at the boundaries between groups, for instance, because they have multiple ancestry components due to admixture. Due to inconsistent groupings when including admixed individuals who share ancestry with multiple groups, allele frequencies of population-specific pathogenic variants may be underestimated. At the same time, the inclusion of admixed individuals with shared ancestry allows us to capture more population-specific pathogenic variants.

To describe the genetic ancestry groups, we used both self-description from individuals in each group as well as their genetic similarity with a reference population (*F*_ST_) (S1 Table). We were able to infer the origins of some founder populations where neither sources of information were available, based on prior reports of disease-causing DNA sequence variants. For example, the *CD55* variant we identified in the Other Jewish/South European group was previously reported in Bukharian Jewish individuals [19]. Genetic ancestry group information is not routinely captured in health records, but may be requested in clinical assessments of rare disease cases. When a clinician seeks to understand a patient’s rare disease, they may request information on detailed family history, seeking evidence of consanguinity and the origins of grandparents. Genetic ancestry group information captured in rare disease care can be used to raise the possibility that known founder variants in that community may be the cause of the affected individual’s rare disease.

The potential that the recognition of the presence of a rare disease in a founder population can lead to targeted therapies is exemplified by the *CD55* variant in the Bukharian Jewish community, which can cause a spectrum of presentations from mild abdominal discomfort following a high-fat meal to a severe syndrome including protein-losing enteropathy, and is effectively treated by the complement C5-inhibitor eculizumab [28]. Implementation in health care systems of information about rare disease susceptibility for founder populations can therefore encompass prenatal screening, clinical decision support to prompt clinicians to be aware of an otherwise rare disease in a patient from a defined community, and can lead to therapeutic interventions.

For a variant to be categorized in ClinVar as Pathogenic or Likely Pathogenic (P/LP), it has to fulfil a number of stringent criteria [29], which may or may not be reliable indicators of pathogenicity. For example, a star system in ClinVar represents the degree of confidence about the variant categorization, reflecting the extent of expert curation of the variant. We note that the *KRT18* variant that meets the criteria for inclusion in **Table 1** is rated with zero stars, and may not be a true risk allele in the European (Ashkenazi Jewish) genetic ancestry group. There are also reasons why categorizations of likely pathogenic or pathogenic variants in ClinVar [15] may not be reliable. For example, older submissions to the database are prone to subsequent conflicting interpretations [30] sometimes because of the failure to appreciate the variant to be relatively common in one understudied population at the time of submission [31]. Our use of ClinVar has revealed many new variants causing rare genetic diseases in under-represented populations of NYC, but we recognize that ClinVar’s bias towards P/LP variants in Europeans implies that there remains even more to be discovered about rare diseases in non-European founder populations.

Additionally, the assumption that variants higher in frequency than the associated disease should be classified as non-causative, may not always be true. Founder populations can have high frequencies of a variant and should be excluded from this filtering approach [32]. This approach is implemented in Grpmax FAF, the filtering allele frequencies offered by gnomAD [33] which excludes founder populations like the Amish, Ashkenazi Jewish and Finnish from frequency calculations. By identifying other founder populations, approaches like Grpmax FAF can be refined with variant frequency information from these additional groups. Another concern is that disease prevalence measurements may vary between communities depending on access to care, which is a concern in NYC [34] and in US health care more generally [35]. If a community lacks access to care, the prevalence of a rare disease in that community may go unrecognized, and likewise an associated disease-causing variant may be misclassified as benign. As we demonstrate here, large-scale population studies like AoU will make it increasingly feasible to gain insights into variant frequencies in different genetic ancestry groups, including founder populations, but some of these genetic ancestry groups will also be defined by limited access to health care. There is the potential to worsen health equity by applying exclusive variant frequency thresholds that fail to recognize the genetic disease burden of founder populations due to inadequate phenotyping. Another potential contributor to health inequalities is undersampling of particular geographic regions in large-scale biobanks like AoU, providing an incomplete picture of rare disease risks across NYC.

We have to balance the value of identifying a genetic disease risk in a community with the risk of stigmatizing that group. The AoU Research Program notes this potential for biased interpretation promoting negative stereotypes [36]. We therefore emphasize how pathogenic variants occur in everyone, regardless of demographic categorization or genetic ancestry (**Fig 3**). What distinguishes founder effect groups is not likely to be the overall burden of genetic damage, but instead the over-representation of specific genetic diseases (**Table 1**) within that burden of damaging variants. We also stress how differences in the numbers of damaging variants in the genomes of people from different parts of the world have more to do with incompleteness of information about, and genomic annotations of, damage. We demonstrate the shortcomings of crude demographic categories such as race and ethnic origin in predicting genetic disease risks. We identified a strong founder effect in the Garifuna ancestry group, but when they self-identify their race and ethnicity they include African American/Black, Hispanic and Latino, and in some cases diverse countries of origin, illustrating the weakness of these categorizations as proxies for genetic variation [2,17]. Similarly, we found multiple self-identified ancestries in some of the non-founder groups, who were not the focus of this manuscript. For example, the ’African’ group included individuals who align with African, African-American, and African-Caribbean ancestry groups, reflected in the wide spectrum of ancestry in the PCA analysis (**Figs 1B and** S1B).

We find that some of the risk alleles from the founder Caribbean populations in NYC also exist at lower frequencies in other Caribbean New Yorkers, because of the complex history of pre-colonial civilization, colonization, slavery and migration. In some cases, individuals of non-Caribbean origin appeared to have founder effect variants that appear in the Caribbean, but these variants were mostly located on shared haplotypes derived from the same continental ancestry (S3 Table). Demography is therefore only modestly informative in predicting disease risk, making any associated stigma tenuous. Instead, we followed the guidelines of the National Academies of Sciences, Engineering, and Medicine (NASEM) on the Use of Race, Ethnicity, and Ancestry as Population Descriptors in Genomics Research [2] to quantify objectively ‘genetic similarity’ using IBD sharing, and ‘genetic ancestry’ labels using those provided by members of each group (S1 Table). We also worked with members of the genetic ancestry groups highlighted in the results to discuss and prepare this report, following recommendation 5 of the NASEM report. These best practice guidelines are clearly of value in using population descriptors in ways that enhance the application of genomic insights in medical care delivery.

This study shows how genetic variants that cause diseases that are rare globally can be common locally within a population, and can influence the spectrum of diseases of patients served by individual health systems. Our focus was on NYC, but the same approaches can be extended nationally using AoU data and comparable international data resources. The insights gained are essential for better health care provision, while highlighting the need to gain insights into the phenotypic manifestations of disease-causing variants in marginalized populations with less access to health care.

## Materials and methods

### Ethics statement

Approval to study these de-identified data was granted by the Albert Einstein College of Medicine Internal Review Board (Protocol 2016–7099). All analyses on this de-identified, secondary data from AoU participants included in the manuscript were also approved by the All of Us Resource Access Board. There were no live participants included in the study.

### Research participants and dataset preparation

Participants living in NYC in the AoU Program version 6 curated data repository [8] were identified by the first three digits of their Zip Code, allowing borough-level resolution of geographic residence. Microarray genotype data were used to assess the population structure of NYC participants by principal component analysis (PCA), by global ancestry analysis as performed by SCOPE [37] and by Identity-by-descent (IBD) analysis as described below. Samples were QCed by call rate and kinship coefficient using PLINK v2.00a2.3LM [38]. No individuals were filtered out by the call rate threshold of 0.9 (---mind 0.1). To remove close relatives, either of the pairs of individuals who showed king kinship coefficients > 0.125 were removed using --king-cutoff 0.125 in PLINK2.0 [38]. Variants of the array data were filtered with the following conditions using PLINK2.0: minor allele frequency >0.01, genotyping rate per site > 0.95, and p-value for the departures from Hardy Weinberg Equilibrium (HWE)> 1x10^-6^ (--maf 0.01 –geno 0.05 --snps-only --hwe 1e-06). After QC steps, 13,817 participants and 720,630 SNPs remained for downstream analysis.

Out of these individuals, 10,381 individuals had whole genome sequence (WGS) data available. We used this subset of individuals and whole genome sequence data from an independent NYC biobank, the Mount Sinai Bio*Me* biobank (dbGaP Accession number phs001644) [9,10], to identify founder pathogenic variants. The IBD analysis was also performed on Bio*Me* dataset to confirm the robustness of our approach and to obtain a reliable reference for population labels. After QC, the Bio*Me* dataset consisted of 11,549 individuals and 982,770 SNPs.

### Comparison of demography between US Census and AoU NYC participants

To show the extent to which our dataset represents the demography of NYC, we compared the proportion of four major self-described race and ethnicities per borough between census data and AoU NYC participants. We obtained census data from the following source: https://www.census.gov/quickfacts/fact/table/richmondcountynewyork,newyorkcountynewyork,queenscountynewyork,kingscountynewyork,bronxcountynewyork,newyorkcitynewyork/PST04522(16).

For AoU NYC participants, self-identified race/ethnicity was obtained using a questionnaire. Participants answered the question: “Which categories describe you? Select all that apply. Note, you may select more than one group.” in the Basics Survey. Borough residence was defined based on the first three digits of the zip code of residence, provided by AoU.

### PCA and global ancestry analysis

PCA and global ancestry analysis were conducted using PLINK 2.0 and SCOPE [37] in supervised mode, respectively, on a combined dataset comprising 13,817 AoU participants and 3,584 individuals from the 1000 Genomes Project (1KGP) [39], the Human Genome Diversity Project (HGDP) [40], and the Simons Genome Diversity Project (SGDP) [41] using a total of 150,213 SNPs. Prior to global ancestry analysis using SCOPE, we conducted ADMIXTURE analysis [42] with K = 5 on this assembled reference panel, and further identified individuals within this panel for whom >95% of their genomes appeared to originate in any of five continental ancestries: African, European, South Asian, East Asian and Native American [39]. The supervised SCOPE analysis was run based on this subset of reference panel participants.

### Identity-by-descent (IBD) analysis

IBD groups, the sets of individuals who share ancestry as defined by shared IBD segments, were constructed from the microarray genotypes. Phasing of the genotypes was conducted with Beagle v5.4 [43] using all populations from 1KGP [39] as references. We used Templated Positional Burrows-Wheeler Transform (TPBWT) [44] on the phased dataset to infer IBD segments >3 cM across all pairs of individuals. The total length of IBD sharing for all pairs of individuals was used to construct an undirected network using the iGraph package [45] in R. To focus on recent demography and to reduce clustering of extended families, we filtered for edges with cumulative IBD sharing ≥12 cM and ≤72 cM, as previously described [11,14]. IBD groups were detected using the infomap.community() [46] function on the constructed network using default parameters. To assess the strength of the founder effect for each IBD group, we estimated the ‘IBD score’, the average length of IBD segments between 3–20 centimorgans (cM) shared between two genomes normalized to sample size, as previously described [12]. We also performed IBD analysis for an independent NYC cohort, the Mount Sinai Bio*Me* Biobank as above and named each Bio*Me* IBD group based on individuals‘ detailed self-reported ethnicity provided separately by Bio*Me* leadership (Alexander Charney, personal communication). To minimize ascertainment bias due to differences in genotyping platforms between the two datasets, we estimated IBD scores for Finns from 1KGP (FIN) using the same set of SNPs for both AoU and BioMe. We then calculated IBD scores relative to FIN. In BioMe and AoU, groups with a relative IBD score greater than 0.5—equivalent to the half of the strength of the founder effect of Finns—were classified as founder populations (**Fig 1C**).

### Inferring ancestral background of individuals in IBD groups

We inferred population ancestry (*e.g.,* Puerto Rican, Dominican, Ashkenazi Jewish) in AoU IBD groups by estimating Hudson’s *F*_ST_ between each group and populations in genomic reference panels using PLINK2.0 and using self-identified information newly added in AoU v8 CDR. The reference panel included 14,985 individuals and 140,952 biallelic SNPs from global populations with sample sizes > 10 individuals in 1KGP, HGDP and SGDP together with IBD groups in Bio*Me*. We also conducted PCA for the merged dataset. IBD groups in AoU and Bio*Me* with *F*_ST_ values < 0.001 were combined in further analyses. *F*_ST_ values between the combined clusters were estimated to see the relationship between clusters and used to write network of IBD populations. The network was plotted with igraph package implemented in R using -log_10_(*F*_ST_) as a distance matrix.

### Detection of pathogenic founder variants for rare diseases

We extracted variants categorized as pathogenic or likely pathogenic (P/LP) in the ClinVar database [15] (version ClinVarFullRelease_2023-01.xml) from WGS data of 10,381 NYC AoU participants and 11,549 Bio*Me* participants. Of the 193,935 P/LP variants registered in ClinVar as of Jan 7, 2023, we detected 27,125 variants in our NYC cohort. We removed close relatives and excluded variants that appeared only once in the NYC dataset. We then filtered variants with genotype rate < 0.9 and p-values for departures from Hardy-Weinberg Equilibrium (HWE) <1x10^-16^. We set a small HWE threshold anticipating that rare variants may likely diverge from HWE due to high heterozygosity. After filtering, 3,616 P/LP variants were observed in NYC individuals. HGVS description and review status (gold stars) were obtained from variant_summary.txt.gz in https://ftp.ncbi.nlm.nih.gov/pub/clinvar last updated on March 30, 2024. The variants which were not classified as P/LP as of March 30, 2024 were removed from results.

We defined seven IBD groups with relative IBD scores > 0.5 as founder populations. To identify founder variants, we set a conservative threshold, including only those that: a) were significantly enriched in a certain founder population compared with other NYC individuals (Fisher’s Exact p < 0.05), b) occurred at a MAF of <0.0001 in NYC individuals not assigned to that group, and c) appeared more than once in that group. We applied the Bonferroni correction (p-value < 0.05/ (3,616 x 7)), but all results are listed in **Table 1** and S2 Table since it is too strict for populations with small sample size. The minor allele frequencies of founder variants were extracted from gnomAD v3.1.225 [47] using gnomAD_DB (https://github.com/KalinNonchev/gnomAD_DB) to compare frequencies in NYC dataset. The number of P/LP variants per individual was also counted for each IBD group. Divergence between populations were assessed by the Wilcoxon rank sum test.

### Ancestry analysis for the founder variant in the Caribbean IBD groups

We identified multiple IBD groups that appeared to have Caribbean ancestry, based on *F*_ST_ analysis against reference populations. Since Caribbean populations have three different continental ancestries in their genomes (African, Native American and European) due to their complex history, we inferred local ancestry around the founder variants detected in Caribbean populations shown in **Table 1** in order to reveal the ancestral background of those founder variants.

Genotype datasets for the carriers were generated by combining the genotype dataset used for IBD analysis and genotype data of each ClinVar variant, and phased by Beagle v5.4 without reference genomes. We then used RFMix [48] version 2 to infer local ancestry ±20 Mb of the variant, with 3 expectation-maximization steps. To assess local ancestry, we assembled a reference panel by identifying individuals from 1 KG, HGDP and SGDP for whom >95% of their genomes appeared to have either African, American or European ancestry based on ADMIXTURE analysis with K = 5 as reference (the same reference individuals in the SCOPE analysis).

## Supporting information

S1 Fig Ancestry background of AoU IBD clusters. (A) PCA plot for all AoU NYC participants and reference panels. (B) PCA plots highlighting individuals belonging to the 14 IBD clusters detected in AoU NYC participants. (C) SCOPE analysis for AoU NYC participants labelled with the 14 IBD clusters. Each color represents global ancestry proportion of the five superpopulations (African, European, American, East Asian and South Asian) inferred using supervised mode in SCOPE. (ZIP)

S2 Fig Comparison of NYC Census data in July 2022 and AoU NYC participants. (A) The proportion of four major races and ethnicities (Asian, Black or African American, Hispanic or Latino and White) in each borough according to the US census (July 2022) and AoU NYC participants. (B) Proportional US census population sizes compared with the proportions of AoU participants from each borough. Although the AoU dataset had disproportionately higher Manhattan and Bronx participants, it showed similar proportions of the four race and ethnicity categories in each borough compared with census data. (TIF)

S3 Fig PCA plot for AoU NYC participants (a), BioMe (b) and global reference populations. PCA plots for (A) AoU NYC participants, (B) BioMe participants, (C) both cohorts compared with global reference populations, and (D) the global reference populations alone. (TIF)

S4 Fig 16 IBD groups in the combined dataset of NYC. PCA plots for the 16 IBD clusters from Fig 1C, with the 7 founder populations highlighted in boxes. (ZIP)

S1 Table Ancestry background assignment to IBD groups. (XLSX)

S2 Table All candidate founder P/LP variants in the seven founder populations in NYC. (XLSX)

S3 Table Frequencies of Caribbean founder variants from Table 1 in other shared ancestry IBD groups. (XLSX)
