# Supplementary figures and images for: Genetic disease risks of under-represented founder populations in New York City
Source: PLoS Genet. 2025 Jun 24;21(6):e1011755. doi: 10.1371/journal.pgen.1011755 (PMC12208467; doi:10.1371/journal.pgen.1011755)

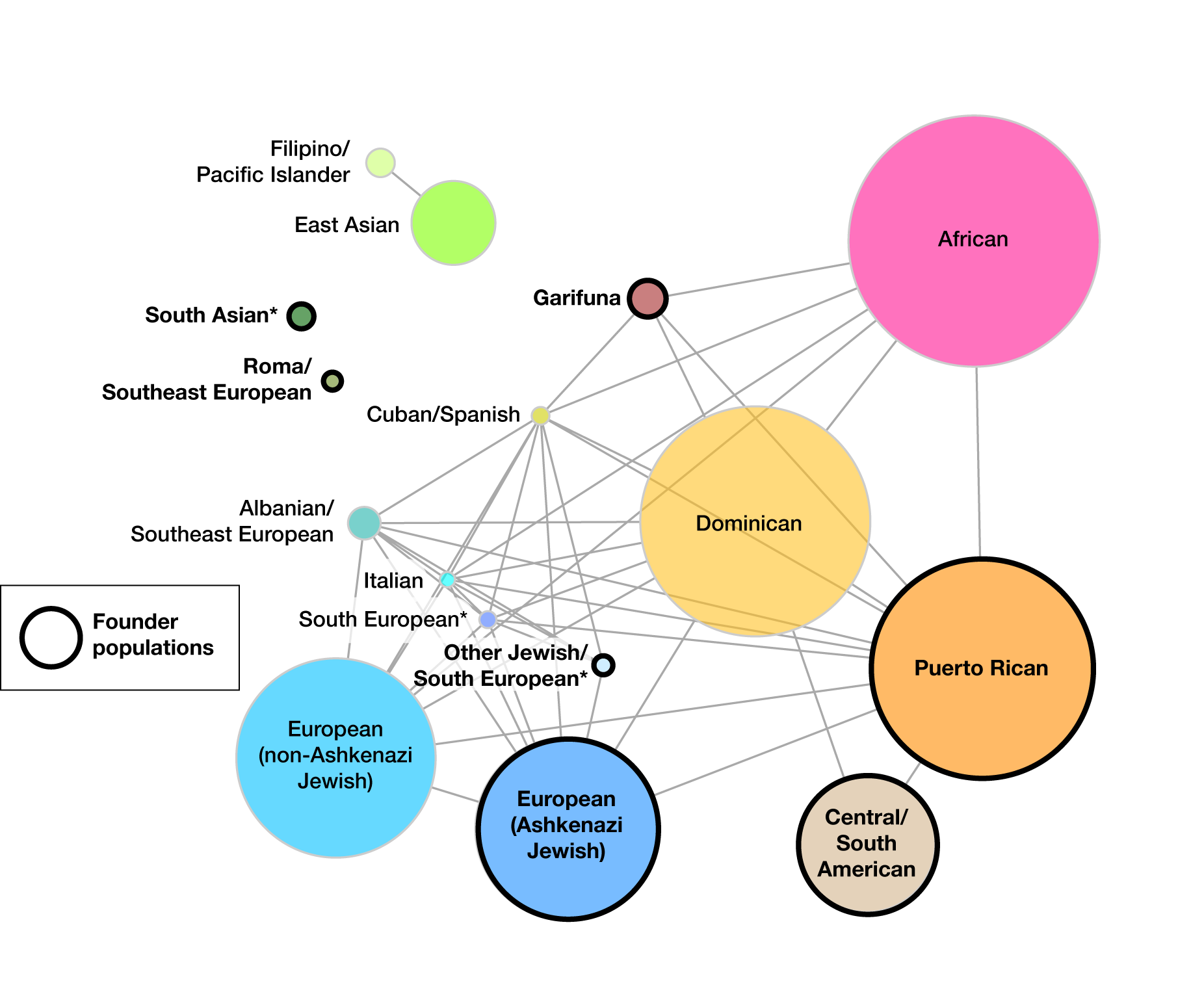

Supplement: S2 Fig — (TIF) [file pgen.1011755.s002.tif]

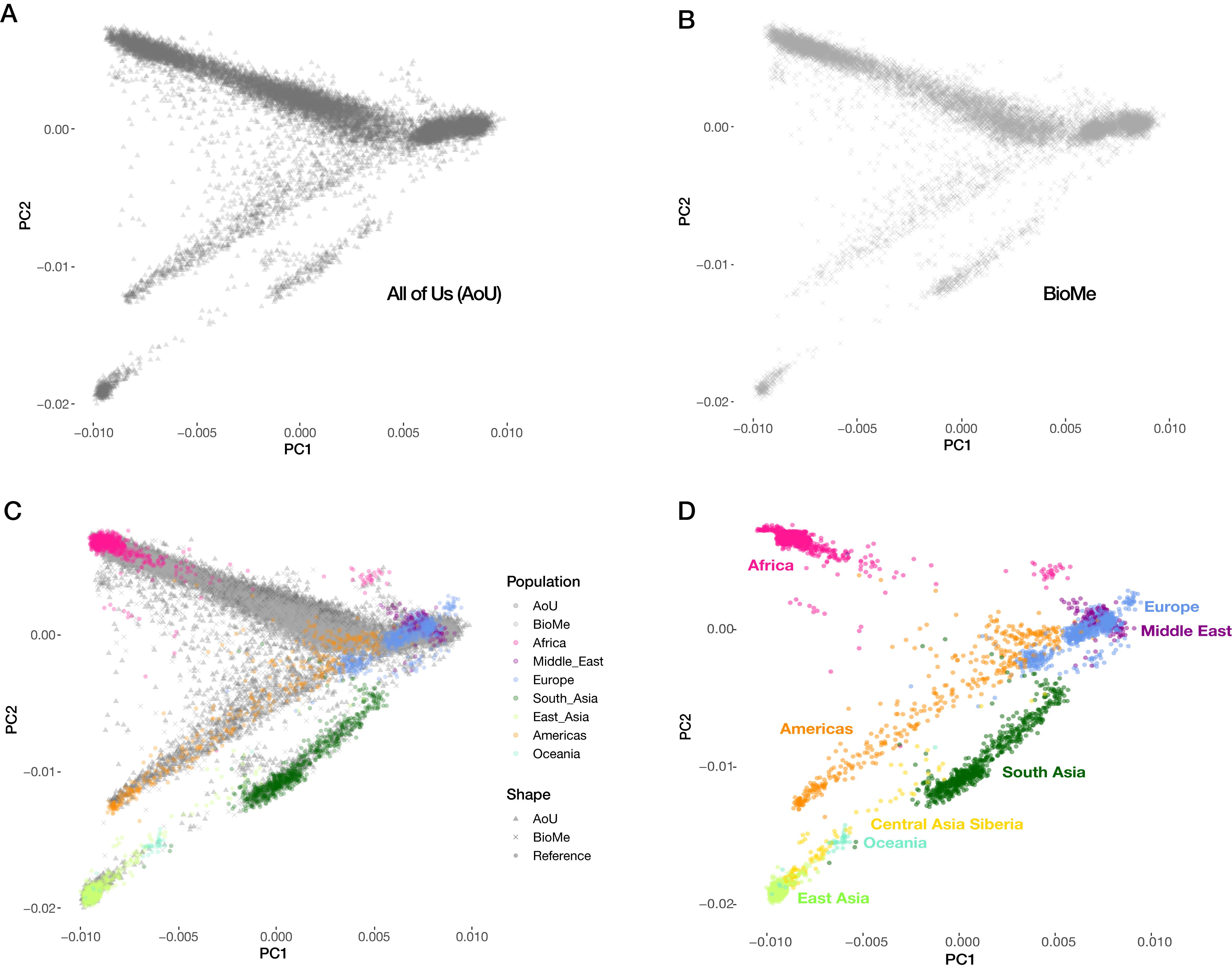

Supplement: S3 Fig — (TIF) [file pgen.1011755.s003.tif]
